# Emergence of Nonfalciparum *Plasmodium* Infection Despite Regular Artemisinin Combination Therapy in an 18-Month Longitudinal Study of Ugandan Children and Their Mothers

**DOI:** 10.1093/infdis/jix686

**Published:** 2018-01-06

**Authors:** Martha Betson, Sarah Clifford, Michelle Stanton, Narcis B Kabatereine, J Russell Stothard

**Affiliations:** 1 School of Veterinary Medicine, University of Surrey, Guildford; 2 Department of Parasitology, Liverpool School of Tropical Medicine, Liverpool, United Kingdom; 3 Vector Control Division, Ministry of Health, Kampala, Uganda

**Keywords:** malaria, *Plasmodium malariae*, *Plasmodium ovale* spp, Uganda, artemisinin combination therapy

## Abstract

As part of a longitudinal cohort investigation of intestinal schistosomiasis and malaria in Ugandan children and their mothers on the shorelines of Lakes Victoria and Albert, we documented risk factors and morbidity associated with nonfalciparum *Plasmodium* infections and the longitudinal dynamics of *Plasmodium* species in children. Host age, household location, and *Plasmodium falciparum* infection were strongly associated with nonfalciparum *Plasmodium* infections, and *Plasmodium malariae* infection was associated with splenomegaly. Despite regular artemisinin combination therapy treatment, there was a 3-fold rise in *P. malariae* prevalence, which was not accountable for by increasing age of the child. Worryingly, our findings reveal the consistent emergence of nonfalciparum infections in children, highlighting the complex dynamics underlying multispecies infections here. Given the growing body of evidence that nonfalciparum malaria infections cause significant morbidity, we encourage better surveillance for nonfalciparum *Plasmodium* infections, particularly in children, with more sensitive DNA detection methods and improved field-based diagnostics.

## Teaser

A longitudinal cohort study of multispecies *Plasmodium* infections in mothers and children in Uganda has revealed there to be both persistent and increasing parasitemia of certain species, which has clinical significance, despite regular access to frontline antimalarial treatments.

Despite progress, control of malaria is a substantial challenge in parts of sub-Saharan Africa [1]. Although *Plasmodium falciparum* is the leading cause of malaria, other species—namely, *Plasmodium vivax*, *Plasmodium malariae*, *Plasmodium ovale curtisi* and *Plasmodium ovale wallikeri—*circulate concurrently, although Duffy-negative individuals curtail *P. vivax* distributions [2–5]. Diagnosis of *P. malariae* and *P. ovale* spp. by light microscopy can be problematic because parasitemias often occur below detection thresholds for expert microscopy or are masked by more visible, concurrent *P. falciparum* infections [6]. Introduction of molecular/serological techniques has revealed that *P. malariae* and *P. ovale* spp. are more common than previously thought [7–11]. In southwestern Uganda, nearly half of asymptomatic children with malaria harbored nonfalciparum species [12].

Despite often being considered benign, a growing body of evidence reports overt disease and morbidity associated with *P. malariae* and *P. ovale* spp. infections [13–17]. In southern Papua, Indonesia, *P. malariae* infection is associated with a high burden of anemia [18, 19], and in Papua New Guinea, where at least 4 *Plasmodium* species cocirculate in humans, detrimental epidemiological interactions occur [20]. A number of other studies have suggested that mixed *P. falciparum*/*P. malariae* infections were associated with increased *P. falciparum* gametocytemia [21–23]. However, evidence of the clinical importance of nonfalciparum *Plasmodium* infections can be conflicting; Black et al demonstrated an inverse relationship between mixed-species infections and fever in Ivory Coast [24], whereas in Nigeria, anemia was shown to be more severe in mixed-species *Plasmodium* infections [25]. In Malawi, Bruce et al concluded that interactions among *Plasmodium* coinfecting species could protect against certain clinical outcomes but was contingent on the local seasonality and intensity of malaria transmission [8].

Commencing field surveillance in 2009 in Uganda, the longitudinal cohort study Schistosomiasis in Mothers and Infants project (SIMI) investigated the dynamics of intestinal schistosomiasis and malaria in young children and their mothers during an 18-month period with regular treatment follow-ups [26]. At baseline, although the general prevalence of noncomplicated *P. falciparum* in children across the 6 SIMI villages was high (>75%), *P. malariae* and *P. ovale* spp. could be found in young children at prevalences of up to 15% and 9%, respectively [27].

In the present study, based on a detailed molecular analysis of the SIMI dried blood spot archive, we identify risk factors for *Plasmodium* species infection, comparing multispecies and single-species infections, and assess interactions among species in terms of clinical outcomes. In Bukoba village, where the prevalence of nonfalciparum *Plasmodium* infections was highest, we conducted a longitudinal and geospatial analysis of all malaria infections that tested for clustering of infection in time and/or space.

## METHODS

### Ethical Statement and Recruitment

The London School of Hygiene and Tropical Medicine, United Kingdom (application no LSHTM 5538.09) and the Ugandan National Council of Science and Technology approved this study. Before enrollment, informed consent was obtained from mothers on their own behalf or on behalf of their children and was documented in writing or by thumbprint (in cases of illiteracy).

### Study Sites, Participants, and Sampling

The longitudinal, closed-cohort SIMI study was conducted in communities of 6 villages on the shores of Lakes Albert and Victoria in Uganda [26]. In total, 662 mothers were enrolled together with 1211 young children (1 or 2 children per mother) aged 5 months to 6 years (49.1% were female). Mothers (or guardians) were aged 15–60 years (see Supplementary Table 1). The SIMI study aimed to investigate the infection dynamics of intestinal schistosomiasis, malaria, and soil-transmitted helminthiases over a period of 18 months, with follow-ups at 6 months, 12 months, and 18 months (Lake Victoria communities only). At each time point, a dried blood spot archive was collected onto Whatman 3M filter paper. A qualified nurse examined each participant on site, carrying out an abdominal examination to assess hepatosplenomegaly and measuring weight, height, and temperature. Each mother was interviewed in the local language to determine their own and their childrens’ exposure to risk factors for infection. The GPS coordinates of study participants’ households were collected as described [28].

### On-Site Diagnosis and Treatment

During each survey, malaria diagnosis was carried out using rapid diagnostic tests (Paracheck-Pf, Orchid Biomedical Systems, Goa, India; or First Response, Premier Medical Corporation, Watchung, NJ) and microscopy on Giemsa-stained blood films [29]. Hemoglobin levels were recorded using a HemoCue spectrometer (HemoCue AB, Angelholm, Sweden). Egg-patent *Schistosoma mansoni* and soil-transmitted helminth infections were also diagnosed on site by microscopic detection of eggs in stool using the Kato-Katz method [26], with diagnosis of intestinal schistosomiasis bolstered by assessing serum antibodies to soluble egg antigen by enzyme-linked immunosorbent assay and circulating cathodic antigen in urine using rapid tests [30].

On the basis of a positive malaria rapid diagnostic test or blood film, children were treated with Lonart (20 mg/120 mg artemether/lumefrantrine; Cipla, Mumbai, India). Praziquantel (40 mg/kg) for treatment of intestinal schistosomiasis was offered to all study participants at baseline and the final survey. For interim surveys, praziquantel was administered on the basis of a positive circulating cathodic antigen urine test. In addition, participants were treated with albendazole (400 mg) at each survey time point. The project nurse supervised all treatment, and participants were monitored for side effects [31].

### Molecular Analysis of Dried Blood Spots

Blood samples on filter paper were stored at 4°C with desiccant prior to genomic DNA extraction using the chelex method [32]. Real-time polymerase chain reaction (PCR) to detect *Plasmodium* species infections was carried out on all baseline samples. *Plasmodium falciparum* infections were detected using a SYBR^®^ green-based real-time PCR assay followed by melt-curve analysis [33]. A probe-based real-time PCR assay [34] was used to detect *P. malariae* and *P. ovale* spp. infections on a Rotorgene RG3000 thermocycler (Corbett, Sydney, Australia). No attempt was made to detect *P. vivax* because analysis of preliminary data demonstrated its absence. For longitudinal analysis of dried blood spots from children in Bukoba village, the probe-based real-time PCR [34] was used to detect *P. falciparum*, *P. malariae*, and *P. ovale* spp. infections at baseline, 6 months, 12 months, and 18 months using the Mx3000P qPCR System (Agilent, Santa Clara, CA).

### Epidemiological and Statistical Analyses

Epidemiological data were analyzed using Stata v9.2 (StatCorp, College Station, TX) and R v2.10.1 (The R Foundation for Statistical Computing, Vienna, Austria). Anemia in children was categorized based on hemoglobin levels as follows: mild, 10–11 g/dL; moderate, 7–10 g/dL; and severe, <7 g/dL. Multispecies malaria infections were categorized as >2 *Plasmodium* species. Intensity of *P. falciparum* infection was either categorized based on Giemsa-stained blood films as high (>5000 parasites/µL) or low (≤5000 parasites/µL) or based on cycle threshold (Ct) values as negative, >40; low, >30–40; medium, >22–30; or high, ≤22 cycles. Univariable regression analysis was performed to identify risk factors associated with *P. falciparum*, *P. malariae*, or *P. ovale* spp. infection as detected by real-time PCR. Factors identified as being statistically associated with a malaria infection (*P* > .05) were incorporated stepwise into a multivariable logistic regression model, and likelihood ratio tests were used to compare models. Random effects were included to control for clustering at household (for *P. falciparum* and *P. malariae*) or village level (*P. ovale* spp.), and interactions among variables were investigated. A similar analysis was carried out to investigate risk factors associated with multispecies malaria versus single-species infections and to investigate associations between morbidity markers and infections with different *Plasmodium* species.

A multiple-kind lottery model lottery-kind model [35] was used to determine whether there was evidence of a departure from random distribution of single and multispecies malaria infections. A generalized linear mixed model with random intercept to account for within-subject correlation was fitted to the time series data from children in Bukoba village to determine the effects of age, time period, and previous infection on current *Plasmodium* infection status.

### Geospatial Analysis

Geospatial analysis of *Plasmodium* species infections among children in Bukoba was carried out based on household GPS locations. Global tests for clustering were undertaken, using the log ratio of spatial densities method proposed by Kelsall and Diggle (1995) to determine whether cases of each species were more clustered than noncases across the village [36]; this testing was conducted using the R package smacpod. This method was also used to identify and map putative local clusters using a Monte-Carlo simulation envelope approach. Coinfection of malaria species at baseline was also examined (ie, *P. falciparum* plus *P. malariae* and *P. falciparum* plus *P. ovale* spp.). There were too few coinfections with all 3 species to explore this spatial structure.

## RESULTS

### Multispecies Malaria Infections

Overall malaria infection prevalence as assessed by microscopy or real-time PCR was substantially raised in children compared with mothers (72.2% [95% confidence interval {CI}, 69.4%–74.8%] vs 24.2% [95% CI, 21.0%–27.7%] by microscopy; 74.9% [95% CI, 72.4%–77.4%] vs 38.6% [95% CI, 34.8%–42.4%] by real-time PCR), and in children infection prevalence was higher in villages along Lake Victoria than along Lake Albert (82.7% [95% CI, 79.5%–85.5%) vs 60.5% [95% CI, 56.4%–64.6%] by microscopy; 82.9% [95% CI, 79.8%–85.8 vs 66.0% [95% CI, 61.9%–69.9% by real-time PCR) (see Supplementary Table 1). *Plasmodium falciparum* was the most common species, with an overall prevalence of 74.6% (95% CI, 72.1%–77.0%) in children and 37.7% (95% CI, 34.0%–41.5%) in mothers. Eighty-nine (7.4%) children were infected with *P. malariae*, and 34 children (2.8%) were infected with *P. ovale* spp. with a higher prevalence along Lake Victoria than along Lake Albert. The majority of children infected with *P. malariae* and/or *P. ovale* spp. were also infected with *P. falciparum*. Only 9 mothers were infected with *P. malariae*, and only 2 were infected with *P. ovale* spp. No individual harbored *P. malariae*/*P. ovale* spp. coinfection in the absence of *P. falciparum*.

**Figure 1.**
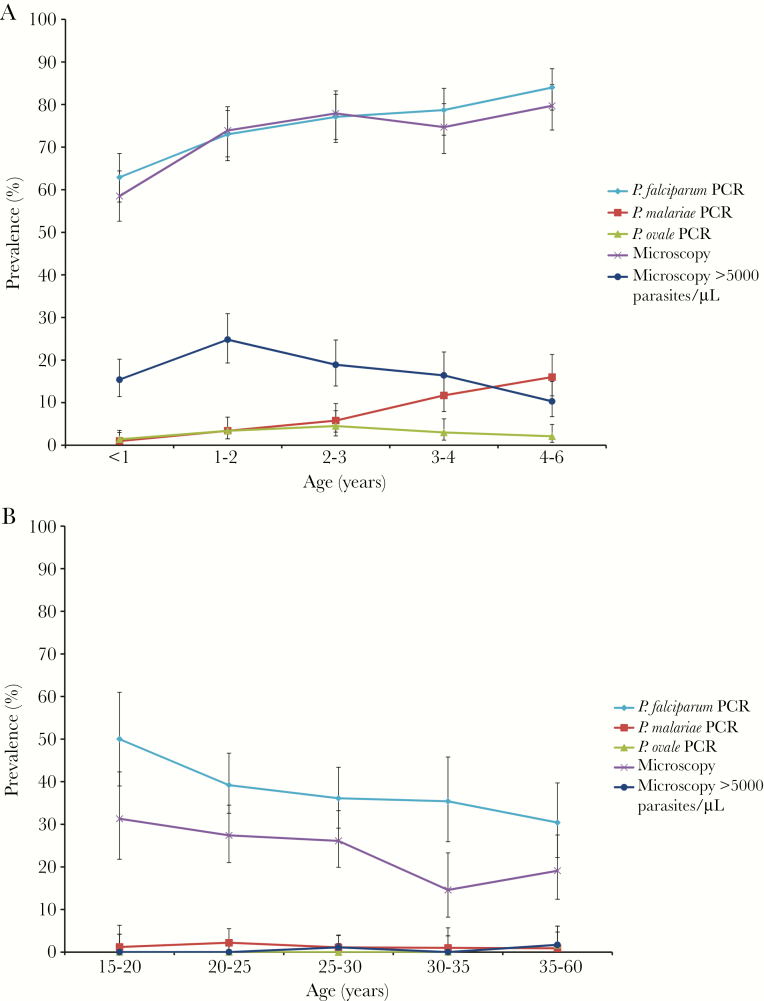
*Plasmodium* infection prevalence in Ugandan lakeshore communities varies with host age. *A*, *Plasmodium* infection prevalence at baseline in children enrolled in the Schistosomiasis in Mothers and Infants (SIMI) study. *B*, *Plasmodium* infection prevalence at baseline in mothers enrolled in the SIMI study. “PCR” refers to infection status determined by real-time polymerse chain reaction performed on DNA extracted from dried blood spots. “Microscopy” refers to presence of parasites in peripheral blood as determined by microscopy on Giemsa-stained blood smears. Error bars represent 95% confidence intervals. Abbreviations: *P. falciparum, Plasmodium falciparum*; *P. malariae*, *Plasmodium malariae; P. ovale, Plasmodium ovale;* PCR, polymerse chain reaction.

The relationship between age and malaria infection prevalence was investigated in children and mothers (Figure 1). The prevalence of slide-positive malaria, *P. falciparum*, and *P. malariae* infections increased with increasing age in children; however the fold difference between the youngest and oldest age category was substantially larger for *P. malariae* infections than *P. falciparum* infections (15.3-fold vs 1.3-fold). In contrast, there was no significant difference in *P. ovale* spp. infection prevalence among the different age groups. The prevalence of highly parasitemic infections (≥5000 parasites/µL) peaked in children aged 1–2 years, then declined in older age groups. In mothers, the prevalence of *P. malariae*, *P. ovale* spp., and highly parasitemic infections was very low and did not vary among age groups. For slide-positive malaria and *P. falciparum* infections, there was a general downward trend in prevalence with increasing age group in mothers. The prevalence of *P. falciparum* infections as detected by real-time PCR was higher than the prevalence of slide-positive malaria, demonstrative of submicroscopic carriage of *Plasmodium* parasites in the mothers.

### Risk Factors Associated With *Plasmodium* Infections

Risk factors associated with *P. falciparum*, *P. malariae*, and *P. ovale* spp. infections in children were investigated using logistic regression analysis. This analysis was not carried out in mothers due to the low prevalence of nonfalciparum infections. In univariable analysis, infection with 1 malaria species was associated with infection with each of the other 2 species and also with hookworm infection (Supplementary Table 2). For both *P. falciparum* and *P. malariae*, there was a strong association with age group, lake system, village, and being inside the house at night. For both infections, owning >1 insecticide-treated bednets (ITNs) was associated with reduced odds of infection, as was sleeping under a bednet in the case of *P. falciparum*. There was also a positive association between *P. falciparum* infection and living in a household with goats or sheep (Supplementary Table 2). *Plasmodium ovale* spp. infection was associated with lake system and living in a household owning goats, sheep, or cows. The final multivariable model for *P. falciparum* included age group, village, *P. malariae* infection, and *P. ovale* spp. infection (Table 1). The model for *P. malariae* was similar but also included hookworm infection and owning an ITN. In contrast, the model for *P. ovale* spp. contained only lake system, *P. falciparum* infection, and *P. malariae* infection (Table 1).

**Table 1.** Multivariable Analysis of Risk Factors for *Plasmodium falciparum, Plasmodium malariae, Plasmodium ovale*, or Multispecies Malaria Infection in Children at Baseline

Species	Variable	Category	Odds ratio	95% CI	*P* value
*P. falciparum*	Age, y	<2	1.00	…	…
		2–4	1.81	1.27–2.57	.001
		4–6	2.71	1.70–4.32	<.0001
	Village	Bugoigo	1.00	…	…
		Walukuba	0.89	.53–1.50	.67
		Piida	1.09	.63–1.90	.76
		Bugoto	2.12	1.25–3.57	.005
		Bukoba	4.01	2.21–7.29	<.0001
		Lwanika	2.11	1.12–3.97	.02
	*P. malariae*	Negative	1.00	…	…
		Positive	7.32	2.10–25.52	.002
	*P. ovale* spp.	Negative	1.00	…	…
		Positive	8.24	.96–70.62	.054
*P. malariae*	Age, y	<2	1.00	…	…
		2–4	6.13	2.49–15.09	<.0001
		4–6	14.82	5.20–42.25	<.0001
	Village	Bugoigo	1.00	…	…
		Walukuba	6.08	1.27–26.10	.02
		Piida	5.03	1.02–24.97	.048
		Bugoto	7.86	1.73–35.71	.008
		Bukoba	12.07	2.56–56.90	.002
		Lwanika	1.74	.30–10.18	.54
	*P. falciparum*	Negative	1.00	…	…
		Positive	7.39	1.95–28.03	.003
	*P. ovale* spp.	Negative	1.00	…	…
		Positive	4.16	1.27–13.57	.02
	Hookworm	Negative	1.00	…	…
		Positive	2.33	.99–5.48	.053
	Houshold owns ≥1 ITN	No	1.00	…	…
		Yes	0.40	.20–.78	.007
*P. ovale* spp.	Lake	Albert	1.00	…	…
		Victoria	5.24	1.28–21.49	.02
	*P. falciparum*	Negative	1.00	…	…
		Positive	6.55	.87–49.10	.07
	*P. malariae*	Negative	1.00	…	…
		Positive	2.98	1.30–6.84	.01
Multispecies	Age, y	<2	1.00	…	…
		2–4	4.64	2.28–9.46	<.0001
		4–6	8.04	3.55–18.22	<.0001
	Village	Bugoigo	1.00	…	…
		Walukuba	2.83	.84–9.53	.09
		Piida	1.49	.40–5.57	.56
		Bugoto	3.42	1.10–10.63	.03
		Bukoba	11.66	3.68–36.92	<.0001
		Lwanika	1.93	.52–7.18	.33
	Household owns	No	1.00	…	…
	≥1 ITN	Yes	0.46	.26–.84	.009

Abbreviations: CI, confidence interval; ITN, insecticide-treated bednet; *P. falciparum*, *Plasmodium falciparum*; *P. malariae*, *Plasmodium malariae*; *P. ovale*, *Plasmodium ovale*.

A similar analysis determined whether there were risk factors associated with multispecies versus single-species malaria infections. In univariable analysis, multispecies infections were associated with age group, lake system, village, hookworm infection, and being inside the house at night. Owning >1 ITNs and sleeping under a bednet were associated with single-species rather than multispecies malaria infections (Supplementary Table 3). The final multivariable model included age group, village, and owning >1 bednets and incorporated random effects to control for clustering at household level (Table 1).

The associations observed among the different malaria species infections (Table 1) suggested that the different species were not randomly distributed. To investigate this in further detail, a multiple lottery-kind analysis was carried out [35]. The numbers of individuals infected with 2 or 3 species were greater than expected, and the number of single-species infections was smaller than expected (Table 2). Overall there was strong evidence of a departure from a random distribution of malaria parasites among infected children (*Χ*^2^ = 33.92; *P* < .0001).

**Table 2.** Multiple-Kind Lottery Model Analysis of the Distribution of Multispecies Infections in Children

Species combination	No. observed	No. expected	*Χ* ^ 2 ^
1 *Plasmodium* species	796	841.84	2.50
2 *Plasmodium* species	101	88.89	1.65
3 *Plasmodium* species	9	1.87	27.21
Not infected	303	276.40	2.56
Total	1209	1209	33.92^a^

^**a**^Degrees of freedom = 3; *P* < .0001.

### Clinical Measures of Malaria

Associations with clinical measures of malaria were then investigated in children. Among parasitemics, no difference in parasitamia between single-species and multispecies infections was detected at either Lake Albert (Wilcoxon’s *W*  =  −0.602; *P*  = 0.182; *N*  = 521) or Lake Victoria (Wilcoxon’s *W*  =  1.33; *P*  = .547; *N*  = 369). In multivariable models, infection with *P. falciparum* was associated with moderate anemia and splenomegaly. In addition, high *P. falciparum* infection levels were associated with fever. Infection with *P. malariae* was associated with an enlarged spleen, and multispecies malaria infections were more strongly associated with spleen enlargement than single-species malaria infections (Table 3).

**Table 3.** Multivariable Analysis of Risk Factors for Various Clinical Indicators of Malaria in Children

Morbidity indicator	Variable	Category	Odds ratio	95% CI	*P* value	*P* value ^ a ^	*P* value ^ b ^
Moderate or severe anemia	*P. falciparum*	Negative	1.00	...	...	...	...
(≤10g/dL)		Low	1.31	.70–2.46	.40	.13	.18
		Moderate	3.58	1.91–6.73	<.0001	.90	.002
		High	5.66	3.04–1.56	<.0001	.20	.02
	Age, y	<2	1.00	...	...	...	...
		2–4	0.19	.08–.36	<.0001	...	...
		4–6	0.48	.08–.41	.14	...	...
	Village	Bugoigo	1.00	...	...	...	...
		Walukuba	0.77	.45–1.33	.35	...	...
		Piida	0.31	.16–.57	<.0001	...	...
		Bugoto	0.19	.10–.33	<.0001	...	...
		Bukoba	0.28	.16–.50	<.0001	...	...
		Lwanika	0.17	.08–.41	<.0001	...	...
	Household	No	1.00	...	...	...	...
	owns ≥1 animal	Yes	0.63	.45–.88	<.007	...	...
	*S. mansoni*	Negative	1.00	...	...	...	...
	(by ELISA)	Positive	0.69	.47–1.00	.050	...	...
Fever	*P. falciparum*	Negative	1.00	...	...	...	...
		Low	0.58	.25–1.37	.22	...	...
		Moderate	0.65	.29–1.44	.29	...	...
		High	2.31	1.22–4.37	.01	...	...
	Age, y	<2	1.00	...	...	...	...
		2–4	1.21	.74–1.99	.44	...	...
		4–6	0.43	.18–1.06	.07	...	...
	Lake	Albert	1.00	...	...	...	...
		Victoria	2.25	1.29–3.92	.008	...	...
	Sleep under a	No	1.00	...	...	...	...
	bednet	Yes	0.53	.33–.84	.008	...	...
Enlarged spleen	*P. falciparum*	Negative	1.00	...	...	...	...
		Low	2.44	1.61–3.68	<.0001	...	...
		Medium	3.94	2.58–6.02	<.0001	...	...
		High	4.92	3.18–7.62	<.0001	...	...
	*P. malariae*	Negative	1.00	...	...	...	...
		Positive	1.81	1.08–3.05	.03	...	...
	Village	Bugoigo	1.00	...	...	...	...
		Walukuba	0.75	.46–1.24	.27	...	...
		Piida	0.53	.31–.92	.03	...	...
		Bugoto	1.13	.70–1.80	.62	...	...
		Bukoba	1.27	.77–2.07	.35	...	...
		Lwanika	1.63	.92–2.89	.09	...	...
	*S. mansoni*	Negative	1.00	...	...	...	...
	(ELISA)	Positive	0.72	.54–.98	.04	...	...
Enlarged spleen	No. *Plasmodium*	1	1.00	...	...	...	...
(multispecies model)	species	>1	1.69	1.04–2.74	.03	...	...
	Village	Bugoigo	1.00	...	...	...	...
		Walukuba	0.84	.47–1.48	.55	...	...
		Piida	0.51	.27–.94	.03	...	...
		Bugoto	1.06	.63–1.79	.83	...	...
		Bukoba	1.21	.71–2.08	.48	...	...
		Lwanika	1.08	.57–2.03	.82	...	...
	*S. mansoni*	Negative	1.00	...	...	...	...
	(ELISA)	Positive	0.63	.45–.88	.006	...	...

Abbreviations: CI, confidence interval; ELISA, enzyme-linked immunosorbent assay; *P. falciparum*, *Plasmodium falciparum*; *P. malariae*, *Plasmodium malariae*; *S. mansoni*, *Schistosoma mansoni*.

^**a**^ *P* value for interaction with age category 2–4 years.

^**b**^ *P* value for interaction with age category 4–6 years

### Longitudinal Infection Dynamics

**Figure 2.**
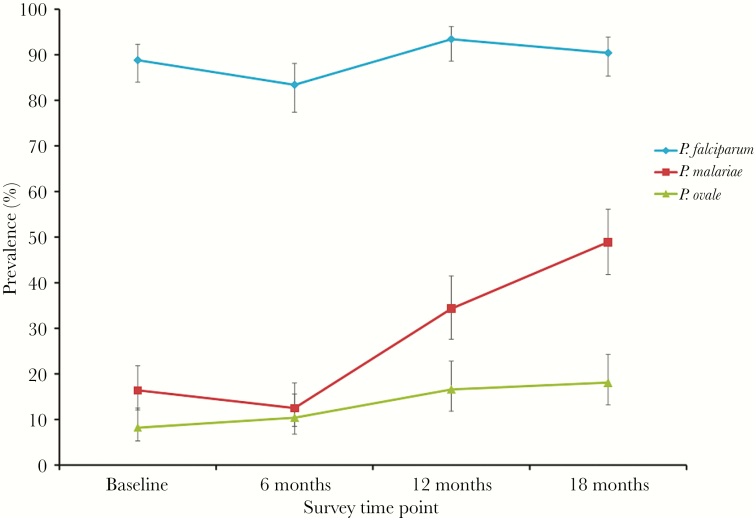
*Plasmodium* infection prevalence in children in Bukoba village at different survey time points. Infection status was determined by real-time polymerase chain reaction performed on DNA extracted from dried blood spots. Error bars represent 95% confidence intervals. Abbreviations: *P. falciparum, Plasmodium falciparum*; *P. malariae*, *Plasmodium malariae; P. ovale, Plasmodium ovale.*

To investigate the temporal dynamics of multispecies malaria infections over the course of the study, the point prevalence of the different *Plasmodium* species infections was determined at 6, 12, and 18 months in children from Bukoba village, where prevalence of nonfalciparum malaria infection was highest [27]. There was a consistent rise in *P. malariae* prevalence (Figure 2), whereas *P. falciparum* prevalance remained largely static and there was a minor upward trend in *P. ovale* spp. prevalence. In the longitudinal multivariable analysis of risk factors, previous *P. falciparum* infection was associated with current *P. falciparum* infection at each time point, whereas mixed *P. falciparum*/*P. malariae* infections were associated with the study time point, the child’s age, and previous *P. malariae* infection (Table 4), demonstrating that the rise in *P. malariae* prevalence was not only due to increasing age of the child. This was supported by stratification of the prevalence of *P. malariae* infection by age group for the different study sites (Supplementary Table 4). Mixed *P. falciparum*/*P. ovale* spp. infections were associated with each time point.

**Table 4.** Longitudinal Analysis of Risk Factors for Infection With *Plasmodium falciparum* (*Pf    * ) Only, *P. falciparum* and *Plasmodium malariae* (*Pf+Pm*), or *P. falciparum* and *Plasmodium ovale* (*Pf+Po*) Among Children in Bukoba Village

Species	Variable	Odds ratio	95% CI	*P* value
Pf only	Previous Pf infection	3.01	1.46–5.83	.002
Pf + Pm	Time point	2.07	1.58–2.84	<.0001
	Age	1.19	1.03–1.41	.03
	Previous Pm infection	2.29	1.21–3.88	.0001
Pf+Po	Time point	1.42	1.16–1.76	.0009

Abbreviations: CI, confidence interval; Pf, *Plasmodium falciparum*; Pm, *Plasmodium malariae*; Po, *Plasmodium ovale*.

### Geospatial Analysis

**Figure 3.**
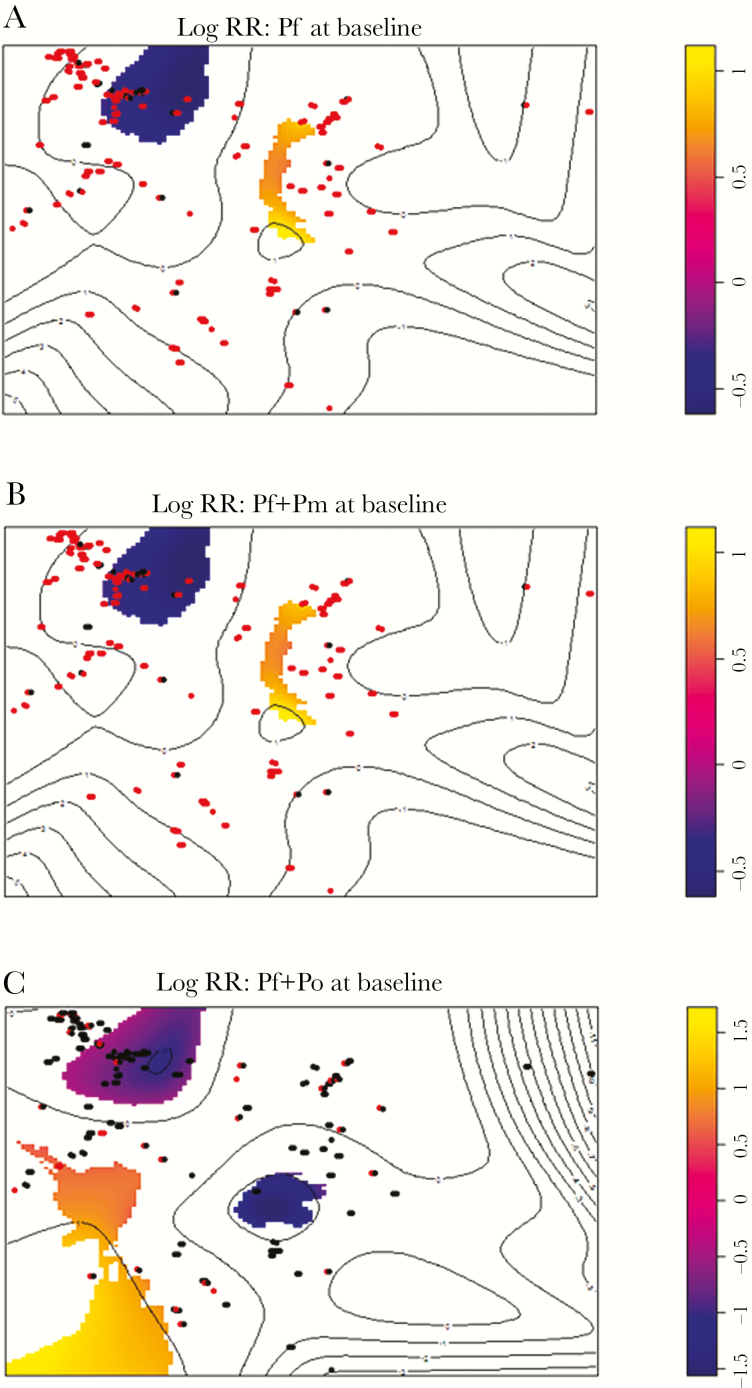
Areas of Bukoba with significant log-transformed relative risk of malaria at baseline, determined using a Monte Carlo simulation envelope approach for *Plasmodium falciparum* infections (*A*), *P. falciparum* and *Plasmodium malariae* infections (*B*), and *P. falciparum* and *Plasmodium ovale* (*C*) infections. Each individual, infected (red) nor not-infected (black), is represented by a dot corresponding to their household. Yellow areas indicate more infections than expected, whereas purple areas indicate fewer infections than expected. Abbreviations: *Pf, Plasmodium falciparum*; *Pm*, *Plasmodium malariae; Po, Plasmodium ovale;* RR, relative risk.

At baseline, there was no obvious visual pattern of infections of any *Plasmodium* species, and global tests for any clustering were nonsignificant (*P* > .05) (Supplementary Figure 1). However, upon examination of maps of significant log relative risk (Figure 3), the area in the northwest of the village appeared to have fewer *P. falciparum*, *P. falciparum*/*P. malariae*, and *P. falciparum*/*P. ovale* spp. cases than expected by chance (*P* = .03).

## DISCUSSION

Our analysis of the SIMI dried blood spot archive has provided a much deeper insight into the complex dynamics and significance of multispecies *Plasmodium* infections in children living in lakeshore communities in Uganda. One of the major risk factors identified for *P. malariae* and mixed-species infections was host age: infection with any *Plasmodium* species was much more common in children than in their mothers, and older children were more likely to be infected with each of the *Plasmodium* species than younger children. This age-prevalence pattern is well established for *P. falciparum* infections, and similar results have been reported for *P. malariae* and/or mixed-species infections in sub-Saharan Africa and Papua New Guinea [8, 11, 37, 38], likely reflecting age-related exposure with partial immunity. The fact that no association was found between *P. ovale* spp. infection and age in our study most likely reflects the low prevalence of *P. ovale* spp. infections in the baseline survey and that our diagnostic approach could not differentiate the 2 subspecies of *P. ovale curtisi* and *P. ovale wallikeri* that have been reported sympatric within this part of Uganda [39].

Infection prevalence by village varied for all *Plasmodium* species and was more common in children along Lake Victoria than along Lake Albert. Children in Bukoba were more at risk of mixed-species malaria infections than elsewhere. Similarly, in Malawi, Bruce et al demonstrated variations in prevalences of different *Plasmodium* species infections among villages [8]. All of our survey villages were located in regions of very high malaria endemicity (entomological inoculation rate > 100 per year) [40], although there is a growing appreciation of local heterogeneities even in high transmission areas, with environmental factors, household factors (eg, inclusive of domestic control measures), and insecticide resistance in *Anopheles* being implicated [41, 42]. Although there was no difference in bednet ownership and use, household construction, and so on among villages (Supplementary Table 1 and data not shown), there are climatic factors that differ between lakes that may influence local anopheline biology [42, 43], with potential (un)favorable local microhabitats alluded to in Figure 3.

We found a strong association between *P. falciparum* infection and *P. malariae* and *P. ovale* species infections, and the vast majority of *P. malariae* and *P. ovale* spp. infections existed as coinfections with *P. falciparum*. Consistent with this, multiple lottery-kind analysis revealed nonrandom distributions of *Plasmodium* species. Other studies have reported a frequency of *P. falciparum* and *P. malariae* coinfections higher than would be expected [38, 44–47], but this literature can be somewhat inconsistent (eg, in Papua New Guinea [48] and in Malawi [8]). Our findings here suggest that there may be common exposures and/or susceptibilities to different *Plasmodium* species, an obvious example of which could be shared *Anopheles* vectors. It is not yet known which vectors play a role in natural transmission of *P. malariae* and *P. ovale* spp. in Uganda [3, 4].

We did not observe any protective effect of mixed-species versus single-species *Plasmodium* infections on any of the clinical indicators of malaria, contrasting with other reports [8, 24], although this might benefit from additional assessments over a longer duration and ascertainment of any other underlying clinical states such as any hemoglobinopathies. Nonetheless, there was an association between mixed *Plasmodium* with *P. malariae* infection and splenomegaly, which, to our knowledge, is the first time this observation has been made and adds to the growing body of evidence supporting the clinical significance in children of nonfalciparum malaria within mixed-species *Plasmodium* infections.

Despite repeated artemisinin combination therapy (ACT) treatments, the dramatic rise in *P. malariae* prevalence seen here is most worrying, notwithstanding an upward trend in *P. ovale* spp. prevalence and consistently high *P. falciparum* prevalence. The rise in *P. malariae* may be partly explained by the increasing age of the children, even though the association between survey time point and mixed *P. falciparum*/*P. malariae* infections was maintained upon controlling for child age. A 4-year longitudinal study of *Plasmodium* infection in children in rural Burkina Faso found a 15-fold increase in *P. malariae* prevalence and a 4-fold increase in *P. ovale* spp. prevalence between 2007 and 2010 [22], an indirect consequence perhaps of drug-induced selection. The latter may also be responsible here in Bukoba because, over the 18-month period, 41% of the children (*N* = 248) received 4 ACT treatments, 27% received 3 treatments, and, based on reporting by mothers, 68% of children received further antimalarial treatment between surveys (unpublished data). Consistent with this hypothesis, we have previously demonstrated the persistence of *P. malariae* infections after ACT treatment in the SIMI cohort, most likely due to recrudescence of parasitemia after treatment rather than relapse per se [27].

In certain settings, it has been argued that *P. malariae* may have a relapsing, hepatic hypnozoite stage analogous to *P. vivax* and *P. ovale* spp. or that there is sequestration of quiescent blood-stage form analogous to an arrested lymphatic stage observed in rodent *Plasmodium* species [49]. This argument is based on historical case reports describing an ability of parasites to persist for decades and on a contemporary evaluation of imported cases of *P. malariae* infection in China, Sweden, and the United Kingdom [50]. The latter study demonstrated a delay in onset to symptoms that ranged from 1 day to 1 year or more and was associated with reported chemoprophylactic use by travelers. Thus the dramatic rise in *P. malariae* prevalence is perhaps a combination of the long-term persistence of *P. malariae* parasite processes and drug-induced selection, alongside implementation of more sensitive methods of molecular diagnosis that go beyond the detection thresholds of expert microscopy.

In conclusion, our findings highlight the cryptic burden of nonfalciparum malaria infections and indicate that there is a potential for emergence of *P. malariae* (and *P. ovale* spp.) infections in the face of frontline treatment for *P. falciparum*. With efforts increasingly directed toward elimination of falciparum malaria, we encourage better surveillance of nonfalciparum *Plasmodium* infections in the future, particularly in children, with more sensitive DNA detection methods and improved field-based diagnostics.

## Supplementary Data

Supplementary materials are available at *The Journal of Infectious Diseases* online. Consisting of data provided by the authors to benefit the reader, the posted materials are not copyedited and are the sole responsibility of the authors, so questions or comments should be addressed to the corresponding author.

Supplementary Table 1

Supplementary Table 2

Supplementary Table 3

Supplementary Table 4

Supplementary Figure 1

## Notes

***Acknowledgments.*** We thank technicians of the Vector Control Division, Ugandan Ministry of Health, for excellent support in the field. We are very grateful to all mothers and children who participated in the SIMI study.

***Financial support.*** This work was supported by the Wellcome Trust (grant 085440) and by the Higher Education Funding Council for England.

***Potential conflicts of interest.*** All authors: No reported conflicts. All authors have submitted the ICMJE Form for Disclosure of Potential Conflicts of Interest. Conflicts that the editors consider relevant to the content of the manuscript have been disclosed.

## Notes

Presented in part: Temporal dynamics of *Plasmodium falciparum*, *P. malariae* and *P. ovale* infection in a high-endemic setting in Uganda. British Society of Parasitology Spring Meeting, Bristol, United Kingdom, April 2013.
