# Supplementary material for: Emergence of Nonfalciparum Plasmodium Infection Despite Regular Artemisinin Combination Therapy in an 18-Month Longitudinal Study of Ugandan Children and Their Mothers
Source: J Infect Dis. 2018 Jan 6;217(7):1099–109. doi: 10.1093/infdis/jix686 (PMC5939692; doi:10.1093/infdis/jix686)
Supplement: Supplementary Table 1 [file jix686_suppl_supplementary_table_1.docx]

**Supplementary Table 1. Summary of characteristics of study participants**

|  | **Lake Albert** | | **Lake Victoria** | | |
| --- | --- | --- | --- | --- | --- |
|  | **Children**  ***N*=572**  **(95%CI)***  **[*n*/*N*]** | **Mothers**  ***N*=332**  **(95%CI)**  **[*n*/*N*]** | **Children**  ***N*=639**  **(95%CI)**  **[*n*/*N*]** | **Mothers**  ***N*=330**  **(95%CI)**  **[*n*/*N*]** | |
| Mean age in yrs (range) | 2.88 (0.4-6) | 28.99 (15-55) | 2.88 (0.4-5) | 28.57 (17-60) |  |
| Female | 49.1 (45.0-53.3)  [281/572] | - | 49.1 (45.2-53.1)  [314/639] | - |  |
| Malaria positive  by microscopy | 60.5 (56.4-64.6)  [339/560] | 22.0 (17.6-26.8)  [72/328] | 82.7 (79.5-85.5)  [520/629] | 26.5 (21.8-31.7)  [87/328] |  |
| Mean log_10_ parasite density per µl blood | 3.22 (3.14-3.30) | 2.67 (2.50-2.85) | 3.10 (3.04-3.17) | 2.39 (2.26-2.52) |  |
| Parasitaemia ≥5000 parasites/µl | 16.7 (13.8-20.1)  [94/560] | 0.9 (0.2-2.6)  [3/328] | 17.3 (14.5-20.5)  [109/629] | 0.3 (0.007-1.7)  [1/328] |  |
| Malaria positive by real-time PCR | 66.0 (61.9-69.9)  [376/570] | 36.0 (30.8-41.4)  [119/331] | 82.9 (79.8-85.8)  [530/639] | 41.2 (35.8-46.7)  [136/330] |  |
| *P. falciparum* (Pf)  positive by PCR | 65.61 (61.6-69.5)  [374/570] | 34.74 (29.6-40.1)  [115/331] | 82.63 (79.5-85.5)  [528/639] | 40.61 (35.3-46.1)  [134/330] |  |
| *P. malariae* (Pm)  positive by PCR | 4.38 (2.9-6.4)  [25/571] | 1.20 (0.3-3.1)  [4/332] | 10.0 (7.8-12.6)  [64/639] | 1.52 (0.5-3.5)  [5/330] |  |
| *P. ovale* (Po)  positive by PCR | 0.7 (0.2-1.8)  [4/571] | 0.3 (0.007-1.7)  [1/332] | 4.7 (3.2-6.6)  [30/639] | 0.3 (0.008-1.7)  [1/330] |  |
| Pf+Pm positive | 4.0 (2.6-6.0)  [23/570] | 0 (0-1.1)  [0/331] | 8.5 (6.4-10.9)  [54/639] | 0.9 (0.2-2.6)  [3/330] |  |
| Pf+Po positive | 0.7 (0.2-1.8)  [4/570] | 0.3 (0.008-1.7)  [1/331] | 3.1 (1.9-4.8)  [20/639] | 0.3 (0.008-1.7)  [1/330] |  |
| Pf+Pm+Po positive | 0 (0-0.6)  [0/570] | 0 (0-1.1)  [0/331] | 1.4 (0.6-2.7)  [9/639] | 0 (0-1.1)  [0/330] |  |
| Schisto-egg patent | 44.5 (40.3-48.7)  [249/560] | 66.8 (61.4-71.8)  [221/331] | 14.4 (11.8-17.4)  [91/632] | 30.6 (25.6-35.9)  [100/327] |  |
| Schisto-CCA | 59.3 (54.9-63.5)  [304/513] | 67.1 (61.7-72.2)  [216/322] | 43.2 (39.2-47.2)  [263/609] | 50.2 (44.6-55.8)  [161/321] |  |
| Schisto-ELISA | 72.0 (68.1-75.7)  [407/565] | 77.4 (72.5-81.8)  [257/332] | 40.8 (37.0-44.8)  [261/639] | 82.4 (77.9-86.4)  [272/330] |  |
| Hookworm | 2.0 (1.0-3.5)  [11/560] | 5.1 (3.0-8.1)  [17/331] | 14.7 (12.0-17.7)  [93/632] | 52.6 (47.0-58.1)  172/327 |  |
| Fever | 3.5 (2.1-5.4)  [19/548] | 1.8 (0.7-4.0)  [6/326] | 9.5 (7.3-12.0)  [60/634] | 3.4 (1.7-5.9)  [11/327] |  |
| Mean haemoglobin in g/dL | 10.1 (10.0-10.2) | 12.4 (12.3-12.6) | 10.7 (10.6-10.9) | 12.8 (12.6-13.0) |  |
| Anaemia –  haemoglobin ≤11g/dL | 68.8 (64.8-72.6)  [393/571] | 17.5 (13.5-22.0)  [58/332] | 51.8 (47.8-55.7)  [331/639] | 10.3  [34/330] |  |
| Severe anaemia –  haemoglobin ≤7g/dL | 3.5 (2.2-5.4)  [20/571] | 0.9 (0.2-2.6)  [3/332] | 3.1 (1.9-4.8)  [20/639] | 0.3 (0.008-1.7)  [1/330] |  |
| Hepatomegaly | 35.0 (31.1-39.1)  [200/571] | 9.4 (6.5-13.1)  [31/329] | 36.6 (32.8-40.5)  [232/634] | 4.8 (2.8-7.8)  [16/330] |  |
| Splenomegaly | 33.3 (29.4-37.3)  [190/571] | 16.7 (12.8-21.2)  [55/329] | 52.2 (48.2-56.2)  [331/634] | 9.4 (6.5-13.1)  [31/330] |  |
| In household owning ≥1 bednet | 72.2 (68.4-75.9)  [411/569] | 71.2 (66.0-76.0)  [235/330] | 76.0 (72.5-79.3]  [481/633] | 75.8 (70.8-80.4)  [248/327] |  |
| In household owning ≥1 ITN | 57.0 (52.8-61.1)  [321/563] | 56.1 (50.6-61.6)  [183/326] | 51.3 (47.4-55.3)  [324/631] | 51.2 (45.7-56.8)  [167/326] |  |
| Sleep under a bednet | 66.8 (62.7-70.6)  [380/569] | 67.0 (61.6-72.0)  [221/330] | 60.9 (57.0-64.7)  [385/632] | 70.6 (65.3-75.4)  [230/326] |  |
| Inside house in night | 21.8 (18.4-25.4)  [122/560] | 16.5 (12.7-21.0)  [54/327] | 85.2 (82.2-87.9)  [541/635] | 88.9 (84.9-92.1)  [287/323] |  |

* 95%CI – 95% confidence interval
