# Supplementary material for: Emergence of Nonfalciparum Plasmodium Infection Despite Regular Artemisinin Combination Therapy in an 18-Month Longitudinal Study of Ugandan Children and Their Mothers
Source: J Infect Dis. 2018 Jan 6;217(7):1099–109. doi: 10.1093/infdis/jix686 (PMC5939692; doi:10.1093/infdis/jix686)
Supplement: Supplementary Table 2 [file jix686_suppl_supplementary_table_2.docx]

**Supplementary Table 2. Univariable analysis of risk factors for *P. falciparum*, *P. malariae*, *P. ovale* infection detected by real-time PCR in children at baseline**

| **Variable** | | **n** | **%** | ***P. falciparum*** | | | ***P. malariae*** | | | ***P. ovale*** | | |
| --- | --- | --- | --- | --- | --- | --- | --- | --- | --- | --- | --- | --- |
|  |  |  |  | **OR** | **95% CI** | ***P*** | **OR** | **95% CI** | ***P*** | **OR** | **95% CI** | ***P*** |
| Gender | Male | 616 | 50.9 | 1.00 | - | - | 1.00 | - | - | 1.00 | - | - |
|  | Female | 595 | 49.1 | 1.17 | 0.90-1.51 | 0.24 | 0.88 | 0.57-1.35 | 0.55 | 1.04 | 0.52-2.05 | 0.91 |
| Age (years) | <2 | 521 | 43.0 | 1.00 | - | - | 1.00 | - | - | 1.00 | - | - |
|  | 2-4 | 453 | 37.4 | **1.70** | **1.28-2.27** | **<0.0001** | **4.48** | **2.27-8.84** | **<0.0001** | 1.65 | 0.78-3.49 | 0.19 |
|  | 4-6 | 237 | 19.57 | **2.53** | **1.71-3.74** | **<0.0001** | **8.84** | **4.43-17.63** | **<0.0001** | 0.91 | 0.32-2.62 | 0.87 |
| Lake | Albert | 660 | 50.2 | 1.00 | - | - | 1.00 | - | - | 1.00 | - | - |
|  | Victoria | 664 | 49.8 | **2.49** | **1.91-3.26** | **<0.0001** | **2.43** | **1.51-3.91** | **<0.0001** | **6.98** | **2.44-19.94** | **<0.0001** |
| Village | Bugoigo | 260 | 19.6 | 1.00 | - | - | 1.00 | - | - | 1.00 | - | - |
|  | Walukuba | 240 | 18.1 | 0.92 | 0.61-1.37 | 0.67 | **4.68** | **1.30-16.85** | **0.02** | 0.37 | 0.37-3.57 | 0.39 |
|  | Piida | 164 | 12.4 | 1.09 | 0.71-1.69 | 0.69 | **4.70** | **1.27-17.37** | **0.02** | -* | - | - |
|  | Bugoto | 268 | 20.2 | **2.02** | **1.33-3.04** | **0.001** | **6.95** | **2.06-23.47** | **0.002** | 1.12 | 0.25-5.05 | 0.89 |
|  | Bukoba | 252 | 19.0 | **3.65** | **2.28-5.83** | **<0.0001** | **12.06** | **3.66-39.75** | **<0.0001** | **6.57** | **1.93-22.34** | **0.003** |
|  | Lwanika | 140 | 10.6 | **2.05** | **1.23-3.40** | **0.006** | 2.77 | 0.65-11.80 | 0.17 | 2.77 | 0.65-11.80 | 0.17 |
| *P. falciparum*  real-time PCR | negative | 307 | 25.4 | - | - | - | 1.00 | - | - | 1.00 | - | - |
|  | positive | 908 | 74.6 | - | - | - | **10.68** | **3.35-34.02** | **<0.0001** | **11.62** | **1.58-85.32** | **0.02** |
| *P. malariae*  real-time PCR | negative | 1121 | 92.6 | 1.00 | - | - | - | - | - | 1.00 | - | - |
|  | positive | 89 | 74.6 | **10.68** | **3.35-34.02** | **<0.0001** | - | - | - | **4.93** | **2.23-10.91** | **<0.0001** |
| *P. ovale*  Real-time PCR | negative | 1176 | 97.2 | 1.00 | - | - | 1.00 | - | - | - | - | - |
|  | positive | 34 | 2.8 | **11.62** | **1.58-85.32** | **0.016** | **4.93** | **2.23-10.92** | **<0.0001** | - | - | - |
| *S. mansoni*  microscopy | negative | 852 | 71.5 | 1.00 | - | - | 1.00 | - | - | 1.00 | - | - |
|  | positive | 340 | 28.5 | 0.89 | 0.67-1.19 | 0.44 | 0.72 | 0.43-1.21 | 0.22 | 0.64 | 0.28-1.49 | 0.31 |
| *S. mansoni*  ELISA | negative | 536 | 44.5 | 1.00 | - | - | 1.00 | - | - | 1.00 | - | - |
|  | positive | 668 | 55.5 | 0.94 | 0.73-1.23 | 0.67 | 1.26 | 0.81-1.96 | 0.31 | 0.80 | 0.40-1.58 | 0.52 |
| Hookworm  microscopy | negative | 1088 | 91.3 | 1.00 | - | - | 1.00 | - | - | 1.00 | - | - |
|  | positive | 104 | 8.7 | **3.08** | **1.63-5.84** | **0.001** | **3.85** | **2.25-6.60** | **<0.0001** | **2.83** | **1.20-6.67** | **0.02** |
| House | Mud/reed/grass | 944 | 78.4 | 1.00 | - | - | 1.00 | - | - | 1.00 | - | - |
|  | Brick/stone/plaster | 260 | 21.6 | 1.23 | 0.89-1.71 | 0.21 | 1.31 | 0.80-2.15 | 0.29 | 1.77 | 0.85-3.67 | 0.13 |
| Roof | Thatched | 840 | 70.1 | 1.00 | - | - | 1.00 | - | - | 1.00 | - | - |
|  | Tin | 320 | 26.7 | 0.94 | 0.70-1.26 | 0.69 | 1.04 | 0.63-1.72 | 0.87 | 1.46 | 0.69-3.08 | 0.32 |
|  | Other | 38 | 3.2 | 1.82 | 0.75-4.41 | 0.19 | 2.52 | 1.01-6.28 | 0.05 | 3.51 | 1.00-12.37 | 0.051 |
| Windows | Open | 1146 | 95.7 | 1.00 | - | - | 1.00 | - | - | 1.00 | - | - |
|  | Screened/glazed | 52 | 4.3 | **4.21** | **1.50-11.77** | **0.006** | 1.36 | 0.53-3.51 | 0.52 | **3.10** | **1.05-9.14** | **0.04** |
| Light | None/candle | 204 | 17.1 | 1.00 | - | - | 1.00 | - | - | 1.00 | - | - |
|  | Oil | 726 | 60.9 | 0.72 | 0.49-1.05 | 0.09 | 0.69 | 0.41-1.16 | 0.17 | **0.36** | **0.17-0.78** | **0.009** |
|  | Electric | 263 | 22.1 | 0.88 | 0.57-1.38 | 0.59 | **0.36** | **0.17-0.76** | **0.008** | 0.37 | 0.14-1.01 | 0.053 |
| Access to tap- or well-water | No | 813 | 68.7 | 1.00 | - | - | 1.00 | - | - | 1.00 | - | - |
|  | Yes | 371 | 31.3 | 1.08 | 0.81-1.44 | 0.59 | 1.17 | 0.74-1.84 | 0.52 | 0.56 | 0.24-1.29 | 0.18 |
| Toilet for household | No | 97 | 8.0 | 1.00 | - | - | 1.00 | - | - | 1.00 | - | - |
|  | Yes | 1109 | 92.0 | 0.91 | 0.56-1.48 | 0.70 | 0.76 | 0.37-1.57 | 0.46 | 0.90 | 0.27-3.01 | 0.87 |
| Household owns goats/sheep | No | 951 | 79.0 | 1.00 | - | - | 1.00 | - | - | 1.00 | - | - |
|  | Yes | 253 | 21.0 | **1.48** | **1.05-2.09** | **0.02** | 1.11 | 0.66-1.87 | 0.69 | **2.72** | **1.36-5.47** | **0.005** |
| Household owns cows | No | 1054 | 87.4 | 1.0 | - | - | 1.00 | - | - | 1.00 | - | - |
|  | Yes | 152 | 12.6 | 1.43 | 0.94-2.19 | 0.10 | 1.32 | 0.72-2.41 | 0.36 | 2.19 | 0.97-4.94 | 0.06 |
| Household owns ducks/chickens | No | 711 | 59.0 | 1.00 | - | - | 1.00 | - | - | 1.00 | - | - |
|  | Yes | 495 | 41.0 | 0.90 | 0.69-1.18 | 0.45 | 0.78 | 0.24-2.57 | 0.69 | 1.28 | 0.65-2.54 | 0.47 |
| Mother knows about malaria | No | 53 | 4.4 | 1.00 | - | - | 1.00 | - | - | 1.00 | - | - |
|  | Yes | 1155 | 95.6 | **1.91** | **1.07-3.38** | **0.03** | 4.29 | 0.59-31.42 | 0.15 | 0.73 | 0.17-3.12 | 0.67 |
| Household owns ≥ 1 bednet | No | 310 | 25.8 | 1.00 | - | - | 1.00 | - | - | 1.00 | - | - |
|  | Yes | 892 | 74.2 | 0.77 | 0.56-1.04 | 0.09 | 0.70 | 0.44-1.11 | 0.13 | 0.72 | 0.35-1.49 | 0.37 |
| Household owns ≥ 1  ITN | No | 549 | 46.0 | 1.00 | - | - | 1.00 | - | - | 1.00 | - | - |
|  | Yes | 645 | 54.0 | 0.77 | 0.59-1.01 | 0.06 | **0.45** | **0.29-0.70** | **<0.0001** | 0.59 | 0.29-1.17 | 0.13 |
| Sleep under a  bednet | No | 436 | 36.3 | 1.00 | - | - | 1.00 | - | - | 1.00 | - | - |
|  | Yes | 765 | 63.7 | **0.71** | **0.54-0.94** | **0.02** | 0.66 | 0.43-1.02 | 0.064 | 0.56 | 0.28-1.11 | 0.10 |
| Inside house at night | No | 532 | 44.5 | 1.00 | - | - | 1.00 | - | - | 1.00 | - | - |
|  | Yes | 663 | 55.5 | **1.65** | **1.26-2.14** | **<0.0001** | **2.30** | **1.42-3.72** | **0.001** | 1.70 | 0.82-3.52 | 0.15 |
| Household uses insect repellents | No | 1149 | 95.8 | 1.00 | - | - | 1.00 | - | - | 1.00 | - | - |
|  | Yes | 51 | 4.2 | 0.88 | 0.47-1.65 | 0.70 | 0.77 | 0.24-2.53 | 0.67 | -* | - | - |
| Disturbed by  mosquitoes | No | 65 | 5.4 | 1.00 | - | - | 1.00 | - | - | 1.00 | - | - |
|  | Yes | 1142 | 94.6 | 1.05 | 0.60-1.86 | 0.86 | 2.60 | 0.63-10.81 | 0.19 | 1.91 | 0.26-14.16 | 0.53 |

* predicts failure perfectly.
