# Supplementary material for: Emergence of Nonfalciparum Plasmodium Infection Despite Regular Artemisinin Combination Therapy in an 18-Month Longitudinal Study of Ugandan Children and Their Mothers
Source: J Infect Dis. 2018 Jan 6;217(7):1099–109. doi: 10.1093/infdis/jix686 (PMC5939692; doi:10.1093/infdis/jix686)
Supplement: Supplementary Table 3 [file jix686_suppl_supplementary_table_3.docx]

**Supplementary Table 3.** **Univariable logistic regression on single/multi-species *Plasmodium* infections in children detected using real-time PCR**

| Variable | Category | n | % | Mixed malaria infection | | |
| --- | --- | --- | --- | --- | --- | --- |
|  |  |  |  | **OR** | **95% CI** | ***P*** |
| Gender | Male | 454 | 50.1 | 1.00 | - | - |
|  | Female | 452 | 49.9 | 0.96 | 0.65-1.44 | 0.86 |
| Age (years) | <2 | 351 | 38.7 | 1.00 | - | - |
|  | 2-4 | 355 | 39.2 | 2.78 | 1.62-4.76 | <0.0001 |
|  | 4-6 | 200 | 22.1 | 4.01 | 2.26-7.10 | <0.0001 |
| Lake | Albert | 376 | 41.5 | 1.00 | - | - |
|  | Victoria | 530 | 58.5 | 2.40 | 1.52-3.79 | <0.0001 |
| Village | Bugoigo | 142 | 15.7 | 1.00 | - | - |
|  | Walukuba | 123 | 13.6 | 2.68 | 0.99-7.28 | 0.05 |
|  | Piida | 111 | 12.3 | 1.76 | 0.59-5.23 | 0.31 |
|  | Bugoto | 206 | 22.7 | 2.85 | 1.13-7.19 | 0.03 |
|  | Bukoba | 217 | 24.0 | 7.14 | 2.8-17.14 | <0.0001 |
|  | Lwanika | 107 | 11.8 | 1.83 | 0.62-5.45 | 0.28 |
| *S. mansoni*  microscopy | Negative | 644 | 72.1 | 1.00 | - | - |
|  | Positive | 249 | 27.9 | 0.74 | 0.46-1.19 | 0.22 |
| *S. mansoni*  ELISA | Negative | 405 | 44.9 | 1.00 | - | - |
|  | Positive | 498 | 55.2 | 1.15 | 0.77-1.72 | 0.50 |
| Hookworm  microscopy | Negative | 800 | 89.6 | 1.00 | - | - |
|  | Positive | 93 | 10.4 | 3.35 | 2.02-5.56 | <0.0001 |
| House | Mud/reed/grass | 700 | 77.6 | 1.00 | - | - |
|  | Brick/stone/plaster | 202 | 22.4 | 1.37 | 0.87-2.16 | 0.17 |
| Roof | Thatched | 629 | 70.2 | 1.00 | - | - |
|  | Tin | 235 | 26.2 | 1.23 | 0.78-1.94 | 0.36 |
|  | Other | 32 | 3.6 | 2.33 | 1.17-6.26 | 0.02 |
| Windows | Open | 851 | 94.7 | 1.00 | - | - |
|  | Screened/glazed | 48 | 5.3 | 1.73 | 0.82-3.68 | 0.15 |
| Light | None/candle | 161 | 18.0 | 1.00 | - | - |
|  | Oil | 531 | 59.3 | 0.54 | 0.34-0.87 | 0.01 |
|  | Electric | 203 | 22.7 | 0.32 | 0.17-0.62 | 0.001 |
| Access to tap or well water | No | 601 | 68.0 | 1.00 | - | - |
|  | Yes | 283 | 32.0 | 0.88 | 0.57-1.7 | 0.57 |
| Toilet for household | No | 74 | 8.2 | 1.00 | - | - |
|  | Yes | 829 | 91.8 | 0.78 | 0.40-1.52 | 0.46 |
| Household owns goat/sheep | No | 699 | 77.5 | 1.00 | - | - |
|  | Yes | 203 | 22.5 | 1.43 | 0.92-2.25 | 0.12 |
| Household owns cows | No | 782 | 86.5 | 1.00 | - | - |
|  | Yes | 122 | 13.5 | 1.51 | 0.89-2.55 | 0.13 |
| Household owns ducks/chickens | No | 537 | 59.5 | 1.00 | - | - |
|  | Yes | 366 | 40.5 | 1.04 | 0.69-1.56 | 0.86 |
| Mother knows about malaria | No | 32 | 3.5 | 1.00 | - | - |
|  | Yes | 873 | 96.5 | 1.35 | 0.40-4.51 | 0.63 |
| Household owns ≥1 bednet | No | 242 | 26.9 | 1.00 | - | - |
|  | Yes | 658 | 73.1 | 0.69 | 0.45-1.06 | 0.09 |
| Household owns ≥1 ITN | No | 427 | 47.6 | 1.00 | - | - |
|  | Yes | 470 | 52.4 | 0.50 | 0.33-0.75 | 0.001 |
| Sleep under a bednet | No | 343 | 38.1 | 1.00 | - | - |
|  | Yes | 558 | 61.9 | 0.61 | 0.41-0.91 | 0.02 |
| Inside house at night | No | 372 | 41.4 | 1.00 | - | - |
|  | Yes | 527 | 58.6 | 1.85 | 1.19-2.85 | 0.006 |
| Household uses insect repellents | No | 864 | 95.9 | 1.00 | - | - |
|  | Yes | 37 | 4.1 | 0.62 | 0.19-2.07 | 0.44 |
| Disturbed by mosquitoes | No | 48 | 5.3 | 1.00 | - | - |
|  | Yes | 857 | 94.7 | 2.14 | 0.65-7.01 | 0.21 |
