# Supplementary material for: Emergence of Nonfalciparum Plasmodium Infection Despite Regular Artemisinin Combination Therapy in an 18-Month Longitudinal Study of Ugandan Children and Their Mothers
Source: J Infect Dis. 2018 Jan 6;217(7):1099–109. doi: 10.1093/infdis/jix686 (PMC5939692; doi:10.1093/infdis/jix686)
Supplement: Supplementary Table 4 [file jix686_suppl_supplementary_table_4.docx]

**Supplementary Table 4. Prevalence of *P. malariae* infection in different age groups at different survey time points**

| **Age**  **(years)** | **Baseline** | | **6 months** | | **12 months** | | **18 months** | |
| --- | --- | --- | --- | --- | --- | --- | --- | --- |
|  | ***N*** | **% (95% CI)** | ***N*** | **% (95% CI)** | ***N*** | **% (95% CI)** | ***N*** | **% (95% CI)** |
| 0-2 | 64 | 1.5 (0.03-8.4) | 51 | 3.9 (0.5-12.5) | 18 | 16.7 (3.6-41-4) | 0 | - |
| 2-3 | 93 | 8.6 (3.8-16.2) | 72 | 9.7 (4.0-19.0) | 67 | 28.4 (18.0-40.7) | 52 | 34.6 (22.0-49.1) |
| 3-4 | 49 | 24.4 (13.3-38.9) | 36 | 22.2 (10.1-39.2) | 32 | 37.5 (21.1-56.3) | 37 | 64.9 (47.5-79.8) |
| 4-5 | 42 | 35.7 (21.6-52.0) | 33 | 21.2 (9.0-38.9) | 32 | 50.0 (31.9-68.1) | 35 | 51.4 (34.0-68.6) |
| 5-6 | 0 | - | 0 | - | 32 | 37.5 (21.1-56.3) | 32 | 53.1 (34.7-70.9) |
| 6 | 0 | - | 0 | - | 0 | - | 32 | 46.9 (29.1-65.3) |
