# Supplementary figures and images for: Emergence of Nonfalciparum Plasmodium Infection Despite Regular Artemisinin Combination Therapy in an 18-Month Longitudinal Study of Ugandan Children and Their Mothers
Source: J Infect Dis. 2018 Jan 6;217(7):1099–109. doi: 10.1093/infdis/jix686 (PMC5939692; doi:10.1093/infdis/jix686)

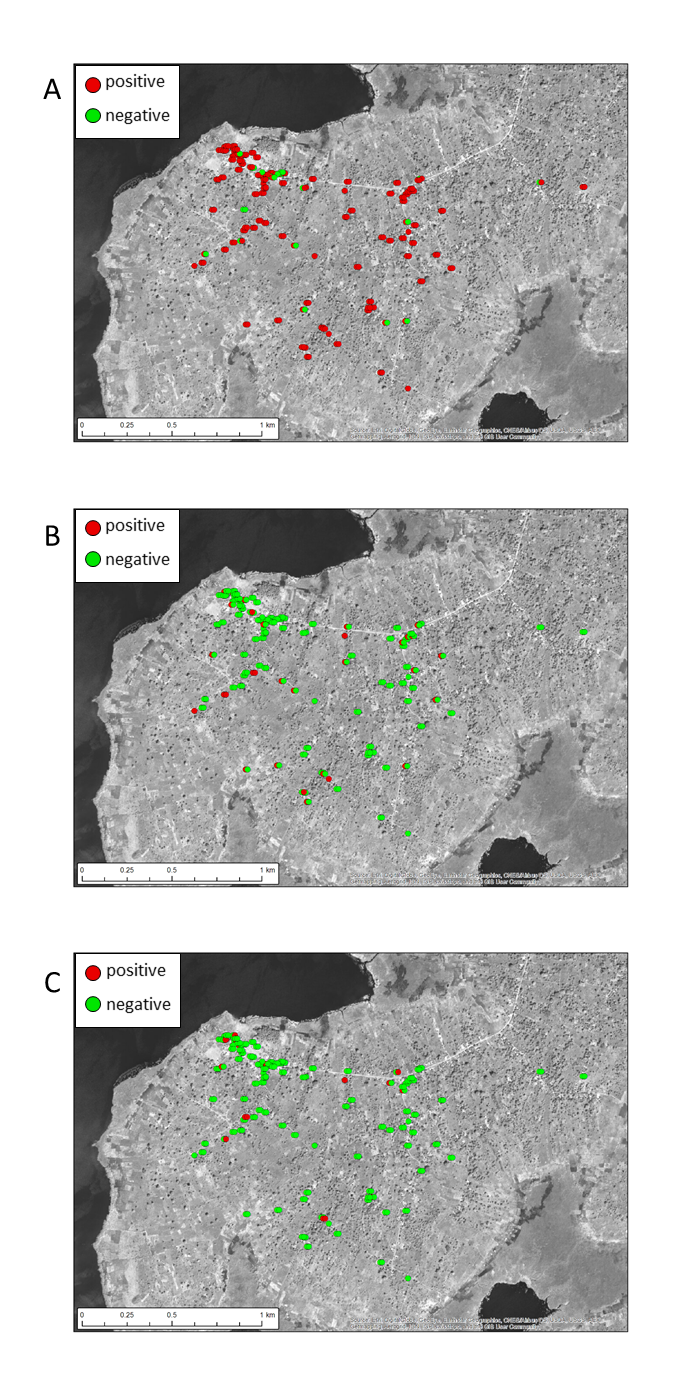

Supplement: Supplementary Figure 1 [file jix686_suppl_supplementary_figure_1.png]
